# Modeling of factors affecting late gadolinium enhancement kinetics in MRI of cardiac amyloid

**DOI:** 10.1186/s12968-023-00952-x

**Published:** 2023-08-10

**Authors:** Leon Axel

**Affiliations:** 1 Department of Radiology, NYU Grossman School of Medicine, 660 First Avenue, Room 411, New York, NY 1016 USA; 2 Department of Internal Medicine, Leon H. Charney Division of Cardiology, NYU Grossman School of Medicine, 660 First Avenue, Room 411, NY 1016 New York, USA

**Keywords:** Cardiac, Amyloid, CMR, MRI, Late gadolinium enhancement, LGE, Contrast kinetics

## Abstract

**Background:**

Late gadolinium enhancement (LGE) is a valuable part of cardiac magnetic resonance imaging (CMR). In particular, inversion-recovery imaging of LGE, with nulling of the signal from reference areas of myocardium, can have a distinctive pattern in some patients with cardiac amyloid, including both diffuse (relatively faint) subendocardial LGE and a relatively dark appearance of the blood. However, the underlying reasons for this distinctive appearance have not previously been well investigated. Pharmacokinetic modeling of myocardial contrast enhancement kinetics can potentially provide insight into the mechanisms of the distinctive LGE appearance that can be seen in cardiac amyloid, as well as why it may be unreliable in some patients.

**Methods:**

An interactive three-compartment pharmacokinetic model of the dynamics of myocardial contrast enhancement in CMR was implemented, and used to simulate LGE dynamics in normal, scar, and cardiac amyloid myocardium; the results were compared with previously published values.

**Results:**

The three-compartment model is able to capture the qualitative features of LGE, in patients with cardiac amyloid. In particular, the characteristic “dark blood” appearance of PSIR images of LGE in cardiac amyloid is seen to likely primarily reflect expansion of the extravascular extracellular space (EES) by amyloid in the “reference” myocardium; the cardiac amyloid contrast enhancement dynamics also reflect expansion of the body EES.

**Conclusion:**

The distinctive appearance of LGE in cardiac amyloid is likely due to a combination of diffuse expansion by amyloid of the EES of the reference myocardium and of the body EES.

**Supplementary Information:**

The online version contains supplementary material available at 10.1186/s12968-023-00952-x.

## Background

**Fig. 1:**
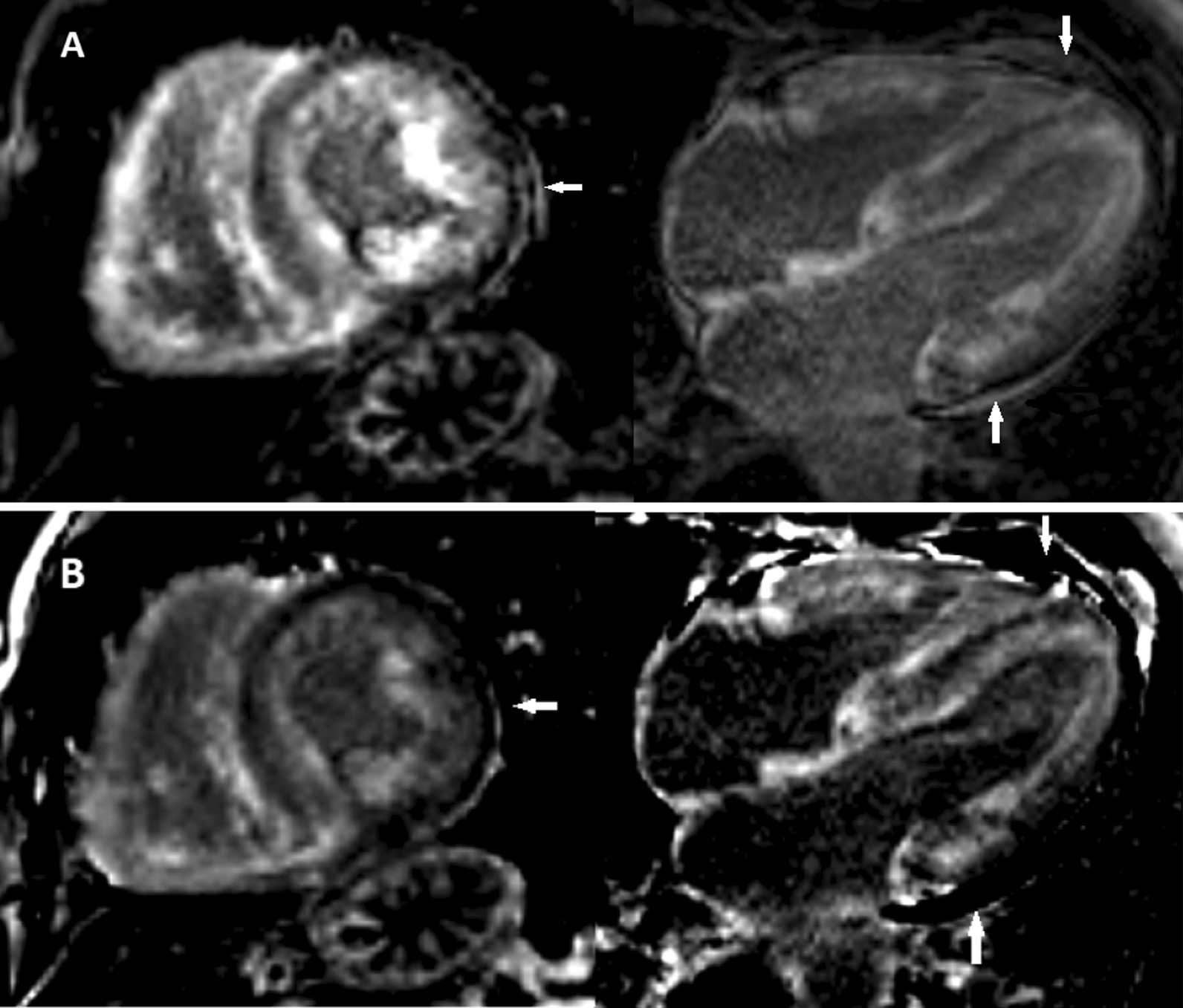
Cardiac magnetic resonance(CMR) imaging with phase-sensitive inversion-recovery (PSIR) of late gadolinium enhancement, in a representative patient with TTR cardiac amyloidosis (left, short-axis view; right, 4-chamber view). The IR timing is adjusted to null the signal from the mid wall of the myocardium, leaving the subendocardium appearing diffusely brighter. The blood pool also appears dark in the PSIR images; this otherwise unusual appearance is common in cardiac amyloid. **A** Magnitude images; note that there is apparent gray signal in regions of a pericardial effusion (arrows), due to rectified negative signal from areas of long-T1 fluid. **B** Phase-sensitive images; note that the fluid regions (arrows) now appear dark, because their signal is lower (negative) than the nulled myocardium

Cardiac involvement in amyloid deposition can lead to heart failure; it can sometimes be difficult to diagnose without biopsy. Cardiac magnetic resonance imaging (CMR) has been reported to be useful in the diagnosis of cardiac amyloid. In particular, as reported by Maceira et al. [1], there can be some qualitatively distinctive features seen in cardiac amyloid, when using late gadolinium enhancement (LGE) CMR with inversion-recovery sensitization to the T1 effects of contrast agent. These distinctive findings include: (1) many patients have diffuse (relatively faint) subendocardial LGE, which may be combined with some mid wall LGE, and (2) there can be loss of the usually pronounced brightness difference between the relatively unenhanced portion of the myocardium and the normally brighter blood pool in the LV cavity (“dark blood”); Fig. 1 demonstrates these features. While cardiac amyloid patients generally also have increased wall thickness and decreased left ventricle function, along with secondary signs of failure, including pleural and pericardial effusions, those findings are not specific for cardiac amyloid. Thus, these distinctive CMR LGE patterns can potentially be of additional diagnostic utility, beyond the usual structural and functional findings of cardiac amyloid in MRI, and they can be used to suggest the possible presence of cardiac amyloid, when seen. To better understand the origins of this distinctive enhancement pattern, Maceira et al. [1] also measured the dynamic effects of contrast agent over time on the T1 relaxation time in blood and the myocardium, in both the cardiac amyloid and control patients; this data was included in their report, and could potentially be used to better understand the underlying pathophysiology. However, the dynamics of contrast enhancement in cardiac amyloid, and its relationship to alterations in the underlying tissue properties, have not previously been specifically investigated with computer modeling of the associated contrast agent concentration kinetics; that is the purpose of the work reported here. Findings on histologic examination of the myocardium of one of the cardiac amyloid patients were also reported in [1]; this information can be used to help guide the modeling of cardiac amyloid myocardium.

It is likely that the more rapid clearance of the contrast agent from the blood pool in cardiac amyloid found in [1] reflects exchange of the contrast agent with an expanded body interstitial extracellular extravascular space (EES), related to systemic amyloid deposition; the renal function would be expected to be decreased, if anything, in cardiac amyloid, which would lead to slower gadolinium clearance through the kidneys. However, this potential mechanism, and its relative contribution to the dark appearance of the blood in LGE imaging of cardiac amyloid patients, has also not previously been investigated with computer modeling of the contrast kinetics.

We have informally observed that there are also some cases where a “characteristic” appearance of cardiac amyloid seen on LGE images in some patients was not confirmed by subsequent myocardial biopsy; on the other hand, there are some other cases where amyloid was incidentally found on a biopsy of the myocardium, but prior LGE images did not show the characteristic appearance of cardiac amyloid (as was also found in some of the AL patients in [1]). The potential mechanisms leading to these cases with discrepant imaging and biopsy findings might also be elucidated through computer modeling of the associated contrast kinetics.

The purposes of this study were to: (1) create an interactive computer program to qualitatively and quantitatively model the dynamics of LGE in the heart, (2) use this program to assess the likely relative contributions of different physiologic factors (particularly amyloid-induced expansion of the EES in the body and in the myocardium) in producing the characteristic LGE appearance of cardiac amyloid, and then (3) use the program to assess some likely reasons why the LGE appearance in CMR and the corresponding presence or absence of cardiac amyloid on biopsy may be discrepant in some patients. This modeling process is challenging, due to the lack of direct knowledge of many of the underlying parameter values and the relatively limited observational data available for estimating them. However, suitable modeling of the LGE dynamics can help to constrain the number of relevant physiologic parameters that need to be considered and their range of likely values [2]. We approach the fitting of the model parameters in a systematic way, in order to reduce the number of degrees of freedom to be considered at any given point in the process. We shall proceed in the spirit of Richard Hamming, who said “The purpose of computing is insight, not numbers” [3].

## Methods

### Observational data on LGE kinetics in cardiac amyloid

As mentioned above, [1] also measured the dynamic effects of contrast agent over time on the T1 relaxation time in blood and the myocardium, in both cardiac amyloid and control patients. While contrast-enhanced T1 was decreased in the subendocardium, compared to controls, it was also decreased in the subepicardium, but to a lesser degree, accounting for the appearance of relatively greater subendocardial enhancement. T1 also increased more rapidly in the blood (described as “more rapid gadolinium clearance” and “faster gadolinium washout” in [1]) in the amyloid patients. The net effect was a decreased difference between the myocardial and blood T1, likely qualitatively accounting for the distinctive appearance in the images of relatively decreased blood signal when nulling the myocardium, in LGE imaging of cardiac amyloid with inversion-recovery methods. These data are thus available for incorporation in a suitable computer model of LGE kinetics.

### Computer model of LGE kinetics

**Fig. 2:**
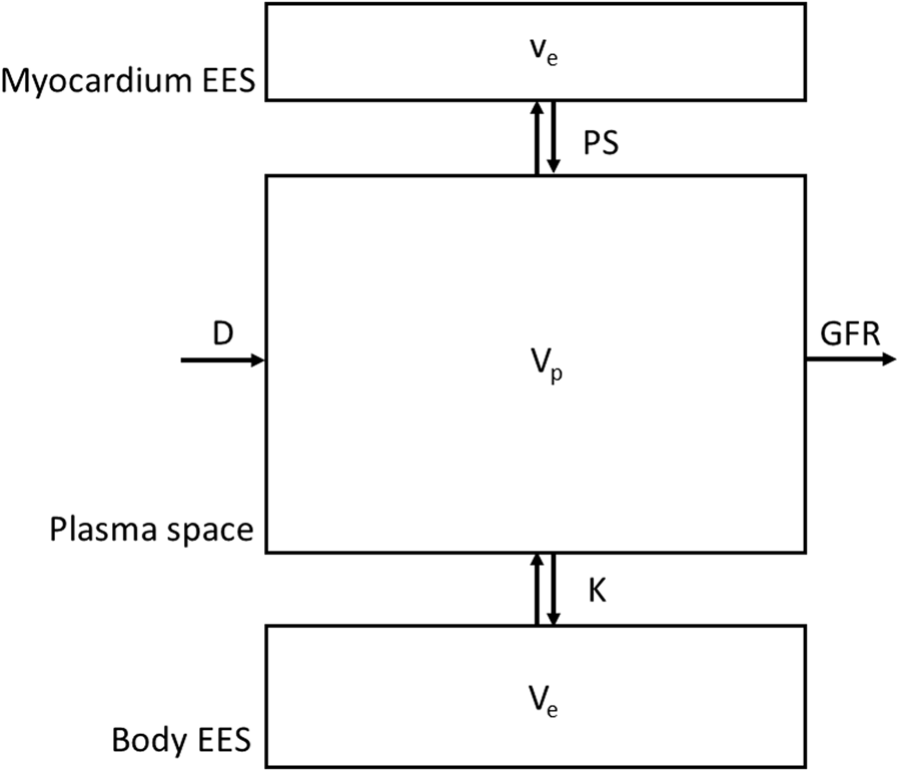
Schematic diagram of 3-compartment model of contrast agent kinetics. Dose, D, of contrast agent is injected into plasma space, with volume V_p_, at initial time = 0. Contrast agent exchanges between plasma and body extravascular extracellular space (EES), with volume V_e_, at a rate dependent on the permeability coefficient of the capillaries between them, K. Contrast agent also exchanges between plasma and myocardial EES, with fractional volume v_e_, at a rate dependent on the permeability-surface area product of the capillaries between them, PS. Contrast agent is cleared from the plasma by the kidneys, at a rate dependent on the glomerular filtration coefficient, GFR

As the characteristic reported changes in cardiac amyloid contrast enhancement are evident by imaging relatively late (minutes) into the enhancement process, we will not attempt to model the more complex effects of the initial redistribution of the contrast agent bolus within the circulating blood (including recirculation), nor the initial “first-pass” myocardial enhancement dynamics (reflecting perfusion). We will assume that the contrast agent distribution is confined to the extracellular space (blood plasma and tissue interstitium). After the initial (relatively transient) events of the distribution of contrast agent around the circulating blood after a bolus injection, the kinetics of the average plasma contrast agent concentration will be predominantly determined by two processes: (1) exchange of contrast agent (driven by concentration differences between them) between the plasma and the body extravascular extracellular space (EES), which can be approximately modelled as a single lumped compartment; and (2) clearance of the contrast agent from the plasma by the kidneys, reflected by the glomerular filtration rate (GFR). The kinetics of the myocardial EES contrast agent concentration will then be predominantly determined by exchange of contrast agent between the plasma and the myocardial EES, initially again modelled as a single lumped compartment. We will also assume that the contribution of the exchange of contrast agent with the myocardium to the overall plasma concentration changes is small, relative to the exchange with the total body interstitial fluid pool, and so we will not model that component explicitly. These assumptions can be represented as a three-compartment model of the contrast agent exchange (Fig. 2). Under the assumption that the plasma space is more strongly coupled to the body EES than the kidneys, the plasma contrast concentration over time after a dose of contrast agent is found to follow a biexponential decay curve, with the myocardial EES following a corresponding triexponential curve, as described more fully in Appendix 1, with the curve parameters dependent on the associated physiologic variables of the exchange model.

The equations for contrast agent concentration over time for the three-compartment model, given in Appendix 1, were used to create an interactive program with MATLAB (Mathworks, Natick, Massachusetts, USA), which can be used to calculate serial blood and myocardial contrast agent concentrations and associated T1 values for given values of the associated model parameters, as described in Appendix 2. The values of the different underlying parameters are not all known or readily determined and may vary between individuals; they may also be affected to varying degrees by diseases, such as cardiac amyloid. However, we can still gain insight into the normal and altered contrast agent dynamics by using initial estimated representative baseline normal values for the parameters in the calculations, drawn from the literature, and then investigating the effects on the calculated blood and tissue contrast kinetics of systematic changes in the different parameter values and comparing the results to published data, in order to derive a reasonably self-consistent set of parameter values, through the fitting process.

As discussed further below, the assumption of a “well-stirred” tissue interstitial space, which is implicit in our use of a 3-compartment model, may not be fully adequate for modeling LGE dynamics of abnormal myocardium with an expanded tissue EES. As an approximate way to capture the effects of the expected spatial inhomogeneity of the myocardial EES in scar and cardiac amyloid, we can represent the myocardial EES as a catenary 2-compartment model (e.g., [4]), as suggested by Moran et al. [5], with the pericapillary component of the myocardial EES exchanging with the plasma, as in Fig. 2, but with that component of the EES also exchanging with a larger but more slowly exchanging remote component of the myocardial EES. This has been incorporated as an option in our computer model of LGE dynamics, as described in Appendix 4.

Initial estimated normal values for permeability-surface area product of the capillaries per unit tissue volume, PS, fractional volume of the myocardial EES, v_e_, and fractional plasma volume of the myocardium, v_p_, were derived from published MRI myocardial perfusion studies [6] and normal T1 values for blood and myocardium were taken from Piechnik [7]. Serial measurements of blood T1 values after a bolus injection of Gd-DPTA are given in [1, 8]. Estimates of plasma volume per unit body weight, V_p_, can be taken from standard physiology texts, as can estimates of EES volume per unit body weight, V_e_, hematocrit, Hct, and GFR, although there is a range of “standard” values given in the literature, and they will depend on age, sex, and body habitus; conventional estimates of blood volume per unit body weight, V_b_, tend to be ~ 75 mL/kg. A value for permeability coefficient of the body capillaries per unit body weight, K, can then be calculated from the formula for m_1_ in Eq. 9 and the value of m_1_ cited above from [9], using the assumed values for V_p_ and V_e_. The resulting set of initial values for the model parameters is shown in Table 1. The associated calculated serial blood and myocardium T1s for a given dose of contrast agent can then be compared with published measurements, e.g., [1, 8], and the initially assumed model parameter values can be empirically interactively adjusted to better fit the observations. Some physical and physiologic constraints on the allowed parameter values, such as hematocrit and EES being less than 100%, are incorporated in the computer model. The resulting set of adjusted model parameters can then be used as a basis for understanding the contrast enhancement dynamics of the normal myocardium, and to explore the results of varying the parameter values in simulations of pathologic states, as discussed below.

**Table 1:** Parameter values derived from the literature and used for initial modeling of normal myocardial contrast kinetics of Gd-DTPA at 1.5 T

D	0.1 mmol/kg
Weight	70 kg
Hct	0.42
Hct_m_	0.30
GFR	90 mL/min (for 70 kg person)
V_b_	75 mL/kg
V_e_	0.15 L/kg
K	0.0083 mL/min
PS_m_	1.3 mL/min
v_e_	0.25
v_mb_	0.09
r1	0.0042 L/mmol/s
T1_b0_	1535 ms
T1_m0_	962 ms

### Modeling of contrast enhancement dynamics of normal myocardium

Although there are a relatively large number of potentially adjustable model parameters, and relatively few data available to constrain them, we can systematically approach the adjustment of their values to fit the model predictions to the observed relaxation times (and their associated contrast agent concentrations), in order to effectively minimize the number of degrees of freedom to be explored at each stage in the fitting process. Extrapolating the observed plasma contrast agent concentration back to the time of the initial bolus injection of contrast agent, before there has been time for any significant exchange of the contrast agent with the EES, allows us to estimate the size of V_p_, using Eq. 1. This value can then be kept fixed for the rest of the calculations. The values of the plasma concentration will then decrease over time, as contrast agent exchanges between the plasma and the EES, approaching a value dependent on the sum of V_p_ and V_e_, at a rate that depends on K. Renal clearance of the contrast agent will lead to a further decrease in the plasma concentration, at a rate dependent on the GFR and the combined volume of plasma and the EES. The associated model parameters can thus be systematically used to adjust the model predictions so as to get a good fit to the observed plasma concentration–time course data. We can take advantage of the different effects of particular parameter value changes on different parts of the time course, in order to partially separate the process of fitting the different parameters associated with the serial plasma concentrations. As the exchange of contrast agent between the plasma and the myocardium is assumed to make a relatively negligible contribution to the overall plasma concentration time course, we can leave these initially adjusted parameter values, associated with the serial plasma concentrations, unchanged while we then adjust the parameters associated with the myocardial contrast enhancement. Again, we can take advantage of the different effects of changes of the different parameter values on different parts of the predicted curves to reduce the number of adjusted values that need to be explored to get a good fit with the data.

In order to make the distribution of the contrast agent within the myocardium more visually apparent in CMR, the associated LGE images are usually acquired with increased T1-weighted image contrast, typically by using inversion-recovery imaging [10]. In inversion-recovery imaging, each set of imaging data acquisitions is preceded by an inversion pulse to invert the tissue magnetization, followed by a delay to allow recovery of the magnetization, which is adjusted to approximately null the magnetization of a selected reference region (typically the most normal appearing part of the heart wall) at the time of the data acquisition (with the specific delay time depending on the T1 value of the region). The reference myocardium will thus appear dark in the resulting images, while other areas with greater concentrations of contrast agent, and associated relative shortening of their T1 times, will have already passed through their magnetization null point and thus appear brighter. One potential problem when using this approach with conventional “magnitude” imaging, is that regions with T1 times longer than the reference region (e.g., in fluid collections) may not yet have gotten to their null point, with associated persistent negative magnetization that can also appear bright in the images (e.g., as is seen in Fig. 1A), as magnitude imaging is insensitive to the sign of the magnetization. To avoid this problem, we can use phase-sensitive inversion-recovery (PSIR) imaging [11], which can use acquisition of a small amount of additional data to correctly reconstruct images of positive and negative magnetization (so that regions of longer T1 will appear darker than the reference region, as seen in Fig. 1B). Raw imaging data acquired with PSIR imaging is typically reconstructed as both magnitude and phase-sensitive images. PSIR imaging is also less sensitive to incorrect setting of the delay time, as the phase-sensitive images will still have correct relative intensities for different regions, related to their corresponding T1 times, even if the reference region is not properly nulled. T1 values calculated with the computer model of contrast enhancement can then be used to calculate corresponding PSIR image intensities, as outlined in Appendix 3.

### Modeling of LGE appearance of myocardial “scar”

The ability to use LGE imaging is one of the strengths of CMR. While not very specific, it is a relatively sensitive way to demonstrate the presence of a wide range of myocardial abnormalities, including post-infarction necrosis and fibrosis, inflammation, infection, interstitial infiltration, and tumor. The basic mechanism of the relatively increased concentration of conventional contrast agent in abnormal myocardium (which leads to the appearance of LGE in the images) is generally considered to be the presence of an expanded extracellular space into which the contrast agent can diffuse, with an associated slowed rate of clearance of the contrast agent from that space [12, 13]. However, this process has been little studied with explicit computer modeling of the underlying myocardial contrast enhancement dynamics. Thus, as an interim step toward modeling contrast enhancement in cardiac amyloid, we can also use the above model of contrast enhancement dynamics to calculate the LGE appearance of a representative area of “scar”, modelling it with an expected expanded EES, v_es_, and potentially a smaller fractional blood volume, v_bs_. For example, Pack et al. [14] found extravascular extracellular volume of 22.9% in normal myocardium and 47.5% in chronic myocardial scar, with myocardial blood volume ~ 0.04 in both; Arheden et al. [15] found extravascular extracellular volume of 23% in normal myocardium and 90% in a rat model of reperfused myocardial infarction. The effective permeability-surface area product of the scar, PSs, will also likely be reduced, reflecting both a smaller net capillary surface area and an overall increased diffusion distance between capillaries and the tissue EES [12, 16]. Note that “diffusion” in this context concerns contrast agent exchange on a tissue microscale of capillaries and the interstitial space between cells; Hedstrom [17] has shown that on a tissue macroscale, diffusion alone is not able to bring significant amounts of contrast agent into areas of myocardium without any perfusion. While specific values for these parameters are likely to be variable between patients (and even between different locations within a given patient), we can use the computer model to qualitatively assess the expected effects of different values of the parameters on the time course of the contrast enhancement.

### Modeling of characteristic LGE appearance of cardiac amyloid

The typical changes observed in the contrast enhancement dynamics in cardiac amyloid [1], relative to the control subjects, as described above, are likely due to cardiac amyloid-associated changes in relevant physiologic factors, which can affect the contrast agent exchange both between the plasma and the body EES, and between the plasma and the myocardium. Amyloid can be deposited in many different tissues throughout the body (to degrees that may vary from patient to patient), effectively expanding the body EES. For patients in heart failure, generalized edema can also expand the body EES, which may also happen with other sources of generalized edema or interstitial fibrosis. Expansion of the body EES, from any source, will tend to decrease the early values of the plasma contrast agent concentration, through effective dilution of the injected dose of the contrast agent into a larger distribution space; however, it will also slow the later clearance of contrast agent by the kidneys from this larger net distribution space. Amyloid damage to the kidneys will decrease the GFR, and thus will also slow the associated late plasma contrast agent clearance. The presence of amyloid would thus not be expected to increase the actual clearance of contrast agent from the body; the appearance of “more rapid gadolinium clearance” found in amyloid patients in [1] likely reflects redistribution into an expanded body EES. While amyloid tends to be preferentially deposited in the subendocardial myocardium, it is typically also fairly diffusely deposited within the rest of the myocardium, thus altering the contrast agent concentration in the “reference” myocardium used in nulling the heart wall in PSIR imaging of LGE. We can use pathology histologic findings (e.g., from [1, 18]) to estimate the degree of expansion of the myocardial EES by amyloid; Maceira’s original report [1] included quantitative histological data from an autopsy of one of the AL patients, with substantial amyloid deposition (30.5% overall), with subendocardial predominance of the amyloid deposition and little fibrosis. The baseline unenhanced (“native”) myocardial T1 is typically increased in cardiac amyloid [19], as is the total myocardial extracellular volume (ECV) (the sum of the tissue plasma volume and EES) [20]. In a study of AL cardiac amyloid patients [20], the myocardial ECV was found to be ~ 0.49 in patients with definite cardiac amyloid (approximately twice the normal value). Although Maceira et al. [1] attempted to get a qualitative estimate of the degree of total body amyloid load with serum amyloid P component (SAP) scintigraphy, they did not find a significant correlation with the contrast agent clearance, which they ascribed to the difficulties of estimating body amyloid with SAP. The expected effects of these various potentially relevant pathophysiologic factors, particularly the degrees of expansion of the EES in the body and the myocardium, and their relative contribution to the typical LGE pattern of cardiac amyloid seen on CMR (in both myocardium and blood), can thus be simulated with the computer model of the contrast agent distribution kinetics, by corresponding adjustments of the associated parameter values.

### Modeling of atypical LGE appearance of cardiac amyloid

The qualitative recognition of the typical appearance of the characteristic LGE pattern in cardiac amyloid (including “dark” blood and diffuse subendocardial LGE) can be used clinically to suggest the presence of cardiac amyloid, when examining CMR images. However, correlation of the CMR appearance with the findings on myocardial biopsy, when available, can sometimes reveal both “false positive” diagnoses, with multiple negative myocardial biopsies found when looking for the presence of amyloid suggested by the CMR appearance, and “false negative” diagnoses, with only nonspecific imaging findings in CMR of patients who are later found to have cardiac amyloid on myocardial biopsy. We can use the computer model of contrast agent kinetics to assess the likely contributions of some clinical factors to such atypical CMR appearances of cardiac amyloid.

## Results

### Modeling of LGE appearance of normal myocardium

**Fig. 3:**
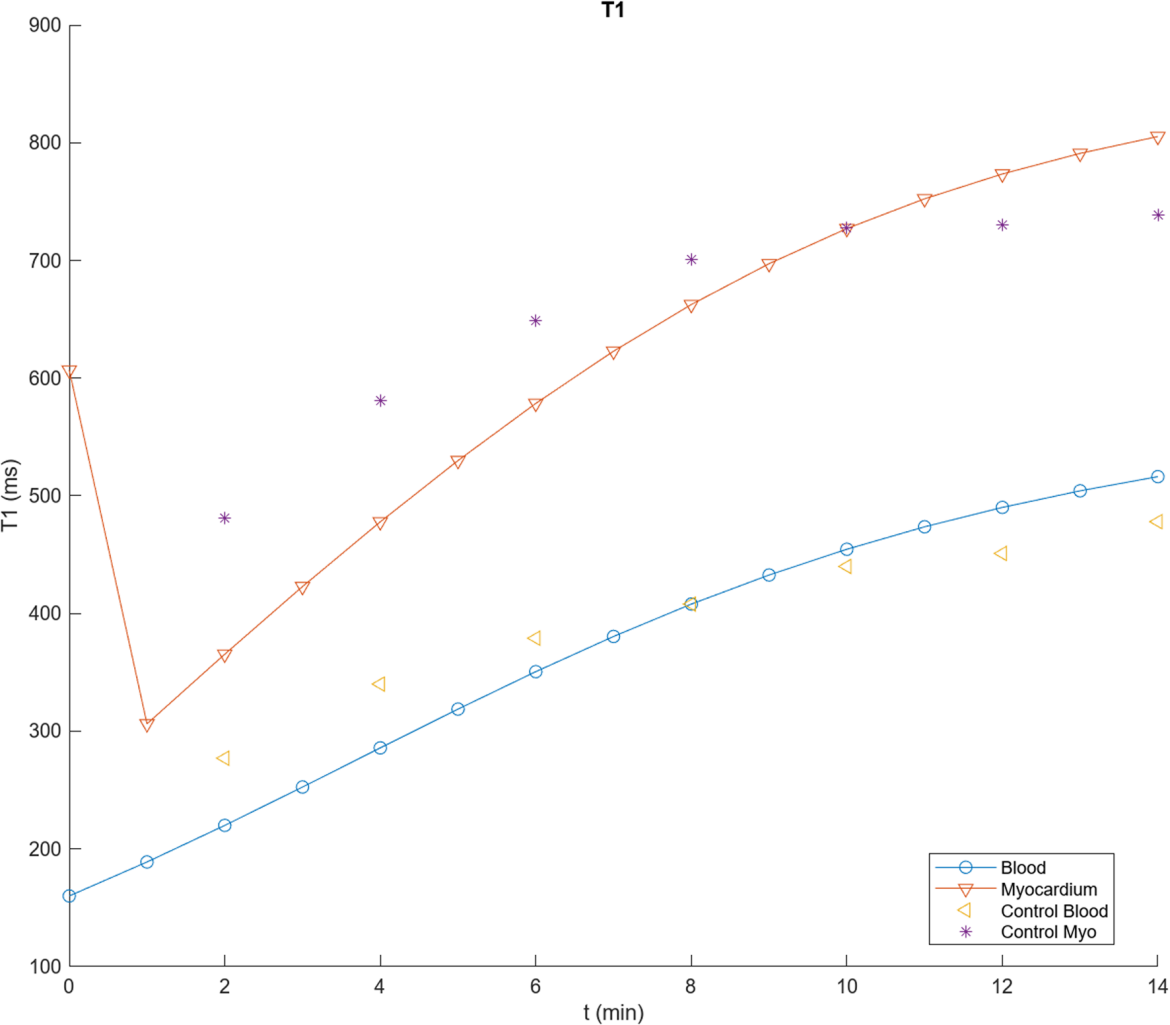
Results of using initial parameter values in Table 1 to calculate T1 values for blood (circles) and myocardium (inverted triangles), plotted together with corresponding data from Fig. 2 of [1] for control subject blood (left-pointing triangles) and myocardium (stars), showing a good fit to the overall scale of the T1 values, but not to the specific shape of the curves

Figure 3 shows the result of calculating the time course of T1 values for blood and myocardium with the program described in Appendix 2, for a dose of 0.1 mmol/kg Gd-DTPA, using the initial parameter values in Table 1 (derived as described above), assuming an average adult, together with corresponding data extracted from their Fig. 2 for the control patients in Maceira et al. [1]. These values can then be systematically empirically adjusted to improve the qualitative agreement between the simulations and the observed contrast enhancement kinetics in normal blood and myocardium. The initial blood T1 value will depend primarily on the total body plasma space, into which the dose is initially diluted; this is seen to be smaller than the observed data extrapolated back to time t = 0. Using relaxivity data from [21] to calculate corresponding contrast agent concentrations, and extrapolating C_b_ to time t = 0, allows us to estimate V_p_ with Eq. 1, assuming a normal hematocrit; these data lead to an estimated V_b_ ~ 0.1 mL/kg; analysis of corresponding blood T1 data from [21] leads to a similar estimate for V_b_. We kept this value for V_b_ fixed during the subsequent adjustments of the other model parameters.

**Fig. 4:**
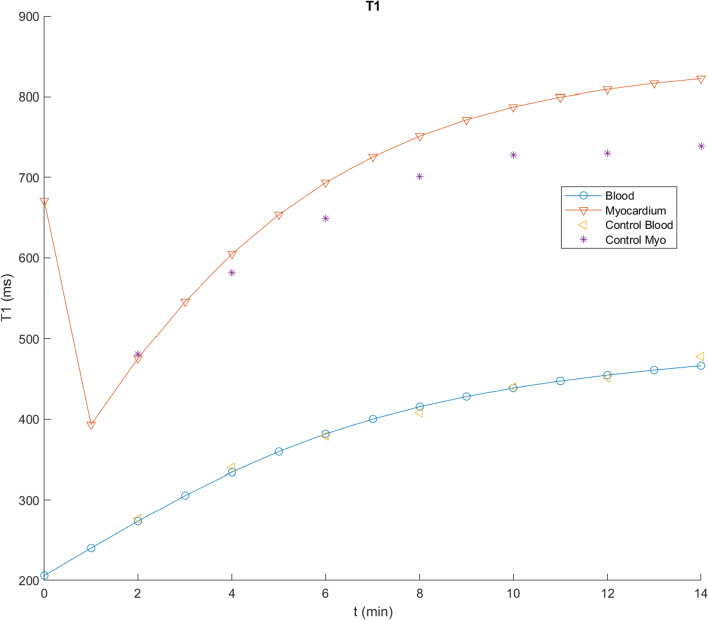
Results of using V_b_ = 0.1 L/kg, K = 0.0108, V_e_ = 0.09, but other parameter values as in Fig. 3, to calculate T1 values, showing an overall good fit to the observed blood contrast kinetics, but residual myocardial fitting errors, particularly for the later times; shown as in Fig. 3

While there is now an improved agreement between the simulated blood T1 and the early observed data, as well as overall good agreement with the rest of the corresponding blood T1 data, there is seen to be some qualitative difference in the shape of the specific time courses, with the initial simulated T1 rising more slowly than the observed early data, but then continuing to increase more rapidly than the later data. The initial part of this T1-time curve depends primarily on the rate of the exchange between the plasma and the body EES, as reflected in K; increasing the value of K by a factor of 1.3, to 0.0108, brings the simulated blood T1 into better agreement with the time course of the early data values. The later part of the curve also reflects the size of the EES; decreasing the value of V_e_ by a factor of 0.6, to 0.09, brings the simulated blood T1 into good agreement with the later data values, as well (Fig. 4). We then kept these adjusted values for V_b_, K, and V_e_ fixed during the subsequent adjustments of other parameters related to normal contrast enhancement dynamics. As expected, changing the value of GFR primarily just affects the later parts of the curves, slowing the late rise in T1 if it is decreased; it was left at the initial value for the subsequent simulations.

**Fig. 5:**
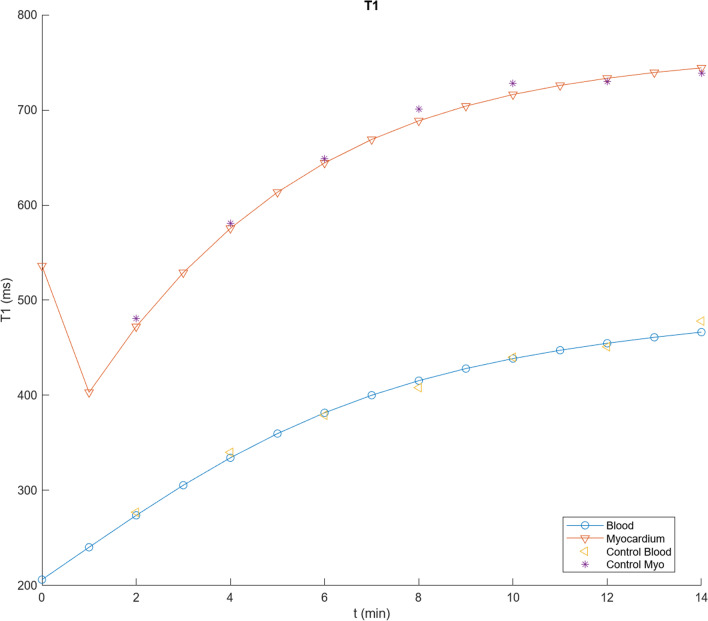
Results of using v_b_ = 0.15, v_e_ = 0.17, PS_m_ = 1.3 and Hct_m_ = 0.24, but other parameter values as in Fig. 4, as summarized in Table 2, showing a further improvement in the fit to the control myocardial T1 values; shown as in Fig. 3

While these parameter value adjustments have improved the fit of the simulations to the control blood T1 data, they have decreased the agreement of the myocardium T1 simulations with the corresponding observed T1 data extracted from Fig. 2 of [1], which are somewhat smaller (higher concentration of contrast agent) and increase more slowly than the initial simulations. The contrast agent in the myocardium is present in the plasma space v_p_ (characterized by v_b_ and tissue hematocrit, Hct_m_) and in the myocardial EES (characterized by v_e_); the exchange of contrast agent between these spaces is characterized by PS of the myocardium, PS_m_. The contrast agent concentration in the myocardial plasma space will just track the blood concentration, with its contribution to the total myocardial contrast agent concentration scaled by v_p_. Similarly, the contribution of the myocardial EES to the total myocardial contrast agent concentration will be scaled by v_e_; increasing v_e_ will decrease the myocardial T1, for a given myocardial EES concentration, C_me_. Thus, in order to lower the myocardial T1 curve calculated with the model, to better match the data, we will need to increase the size of the myocardial extracellular space and/or increase the contrast agent concentration in the myocardial EES. The size of the myocardial extracellular space in the model can be increased through some combination of raising v_e_ and v_b_ and lowering Hct_m_. Increasing the value of PS_m_ will speed the initial entry of contrast agent into the myocardial EES, but it will also speed the later clearance from the myocardial EES as the plasma concentration drops. Empirically adjusting the relevant parameters for the myocardial enhancement to be v_b_ = 0.15, v_e_ = 0.17, PS_m_ = 1.3, and Hct_m_ = 0.24 gives a good fit to the control myocardium values in [1] (Fig. 5). The equivalent value for the combined extracellular volume of the myocardium is ~ 0.29 for these parameter values; this is comparable to the values found by using T1 mapping before and after contrast agent administration [22]. While the particular results found will depend on the specific choices of parameter values, which can be interactively adjusted in the program, it can be seen that the qualitative features of the myocardial and blood contrast enhancement kinetics with these adjusted parameter values are overall comparable to the data from the control subjects in [1]; these qualitative features are not very sensitive to the specific choices of parameter values. Thus, the predictions of this set of parameter values for contrast enhancement of control myocardium are close enough to the observed data to be able to use them as a starting place for modeling of the enhancement of abnormal myocardium. The final set of modified parameter values found after the iterative adjustments above for modeling normal myocardial enhancement is shown in Table 2.

**Table 2:** Modified parameter values from Table 1, after iterative adjustment for improved modeling of normal myocardial contrast kinetics of Gd-DTPA at 1.5 T

D	0.1 mmol/kg
Weight	70 kg
Hct	0.42
Hct_m_	0.24
GFR	90 mL/min (for 70 kg person)
V_b_	100 mL/kg
V_e_	0.09 L/kg
K	0.0108 mL/min
PS_m_	1.3 mL/min
v_e_	0.17
v_mb_	0.15
r1	0.0042 L/mmol/s
T1_b0_	1535 ms
T1_m0_	962 ms

### Modeling of LGE appearance of myocardial “scar”

**Fig. 6:**
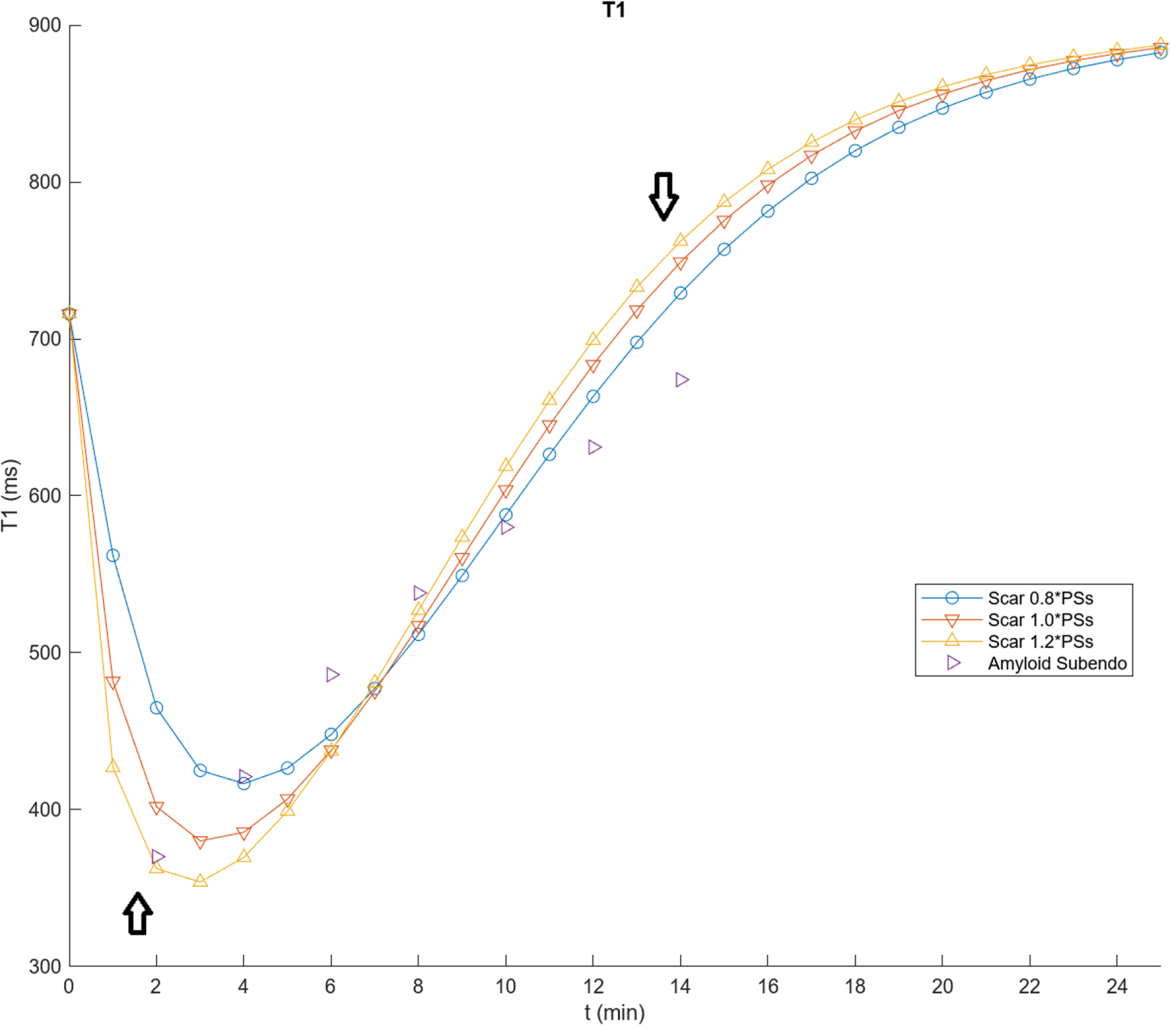
Results of using a range of modified values of PS_s_, scaled relative to PS_s_ = 0.4, and other values from Table 3 (to simulate scar), to calculate T1 values (here labeled as “scar”); shown as in Fig. 4, with addition of subendocardial amyloid T1 data taken from [1] as a rough qualitative comparison (lacking comparable available scar data). Note how increasing PS_S_ produces faster early enhancement (left arrow), but also faster washout of the contrast agent (right arrow)

For initial simulation of the LGE dynamics of a typical myocardial enhancing “scar”, such as can result from prior myocardial infarction, we increased the corresponding value of the scar EES, v_es_ (to ~ 0.95); decreased the scar permeability-surface area product, PS_s_, (to ~ 0.4); decreased the scar blood volume, v_bs_, (to ~ 0.05); and increased the scar native T1, T1_s_0, to 1000 ms, as summarized in Table 3. To illustrate the sensitivity of the fitting process to the parameter values, representative modelling results found by using these values are shown in Fig. 6, for three different values of PS_s_; subendocardial myocardial T1 values from cardiac amyloid patients in Fig. 2 of [1] are also shown for rough comparison. Note that while the overall scale and time course of the modeled values are similar to the patient data, the shape of the curve is somewhat different, with a slower appearance of the contrast agent in the tissue and a faster clearance from it. Increasing the value of PS_s_ will increase the early entry of contrast agent into the scar, but will also speed the washout of contrast agent from it later. Adjusting the values of v_e_ and v_b_ can be used to adjust the scale of the curves, but they are limited by physical constraints to sum to less than 1.0. Also note that the observed data on subendocardial LGE in cardiac amyloid patients would also reflect the influence of contrast agent exchange with an altered body EES, which is not included in the adjustment of the modeling parameters related to the exchange with the myocardium alone.

**Table 3:** Modified parameter values used to simulate myocardial scar or subendocardial amyloid-infiltrated myocardium contrast kinetics

PS_s_	0.41 mL/min
v_es_	0.95
v_bs_	0.05
T1_s0_	1000 ms

**Fig. 7:**
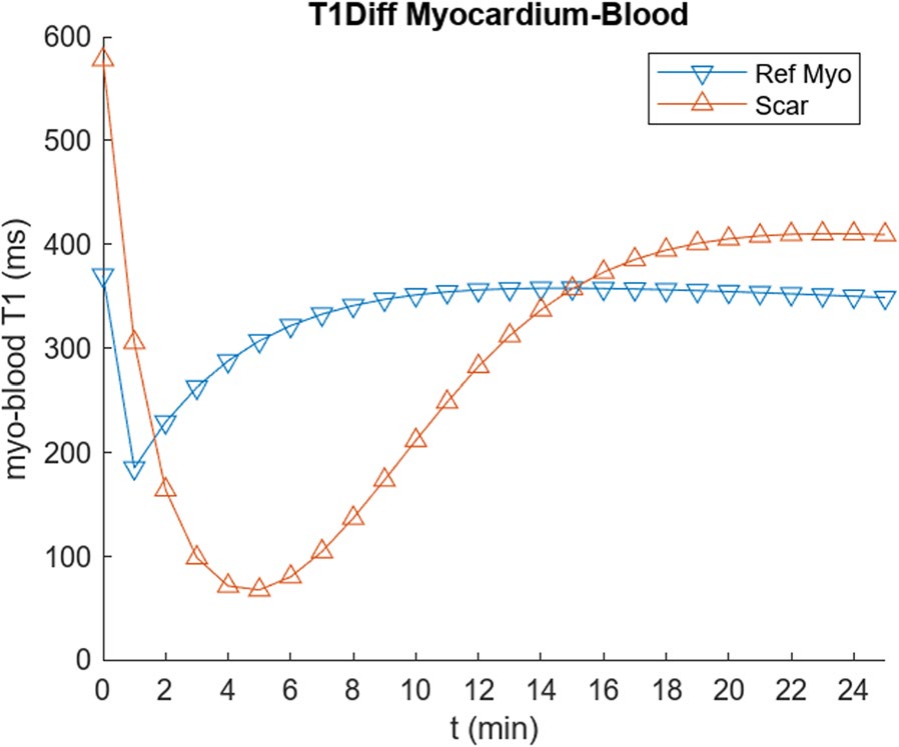
Calculated difference of blood and myocardium T1, for normal myocardium and simulated scar, using parameter values in Tables 2 and 3, respectively

**Fig. 8:**
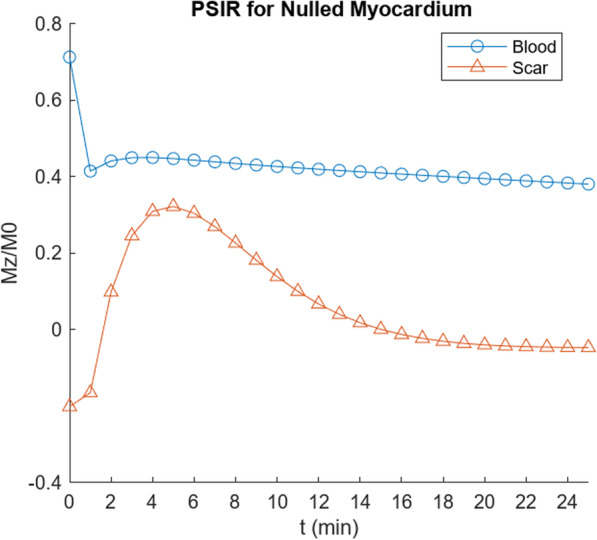
Calculated signal for PSIR imaging with nulling of reference myocardium, for simulated scar (parameter values as per Table 3), showing early peaking of scar enhancement signal and slowly falling blood signal, as expected from clinical observations

Corresponding calculations of the difference of the T1 of the blood, and the T1 of the normal and the simulated scar myocardium, are shown in Fig. 7, for comparison with the data in [1]. Calculation of the corresponding PSIR signal is shown in Fig. 8. Note that while the curves settle into a similar relationship at later times, this “pseudo steady-state” is not a true steady-state, due to continued renal clearance of the contrast agent.

### Modeling of “typical” LGE appearance of cardiac amyloid

**Fig. 9:**
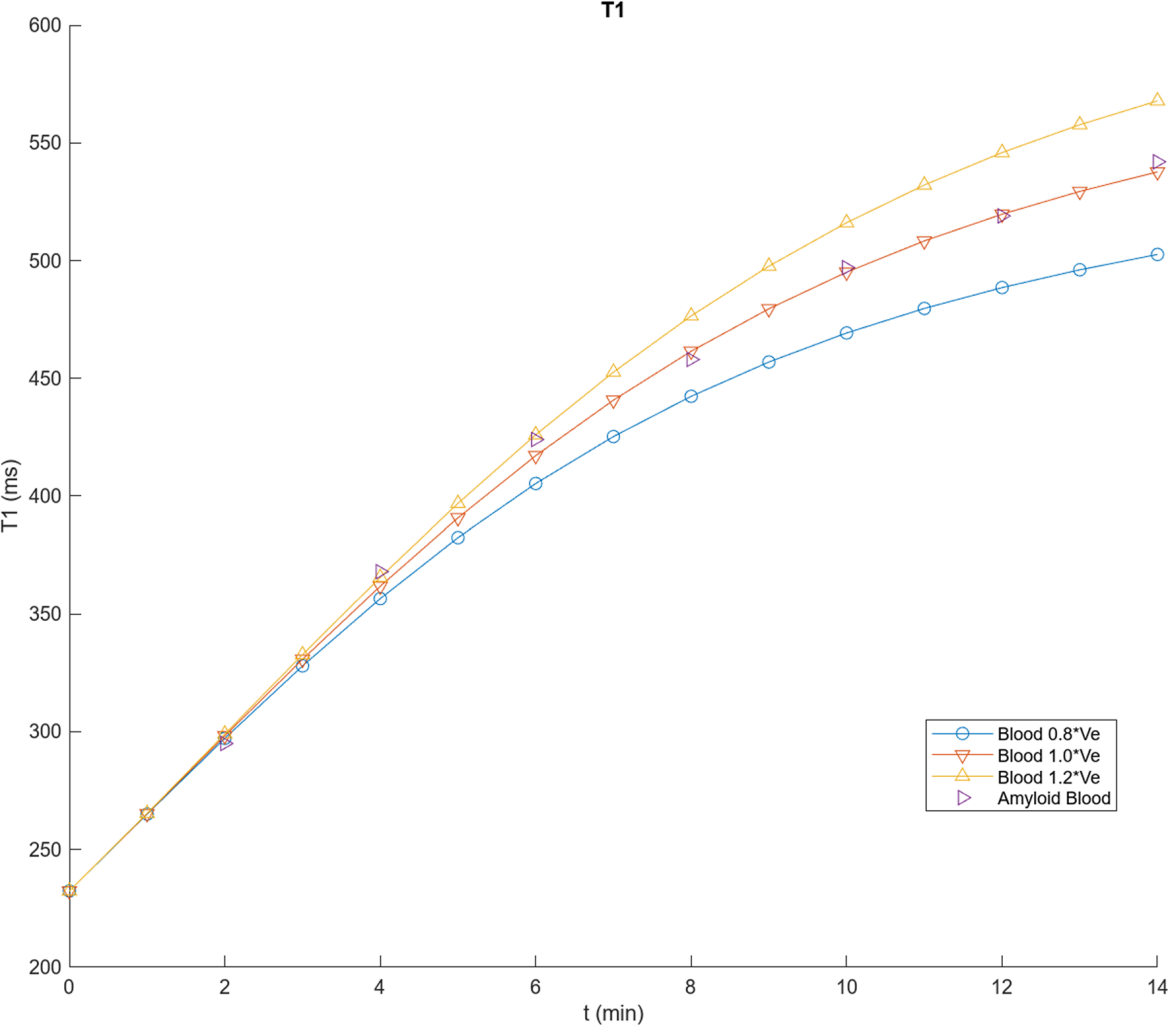
Results of simulation of blood T1, using V_b_ = 0.115 and three values of V_e_, scaled relative to V_e_ = 0.13, with addition of amyloid blood T1 data extracted from Fig. 2 of [1], showing a good fit for V_e_ = 0.13

**Fig. 10:**
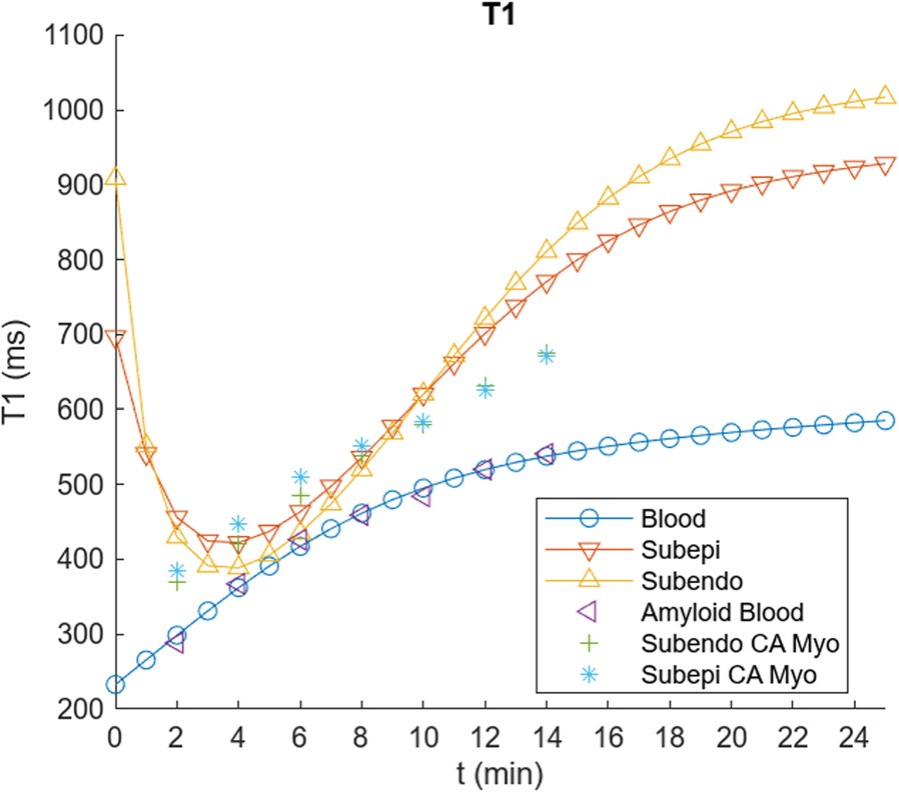
Results of using modified values from Table 4 to simulate cardiac amyloid effects on reference myocardium and body EES, but other parameter values as in Fig. 9, to calculate T1 values; shown as in Fig. 4, with addition of subepicardial and subendocardial amyloid T1 data from Fig. 2 of [1], showing a good overall fit to the scale of the data but an imperfect fit to the specific shape of the early and late phases of the myocardial enhancement curves

**Fig. 11:**
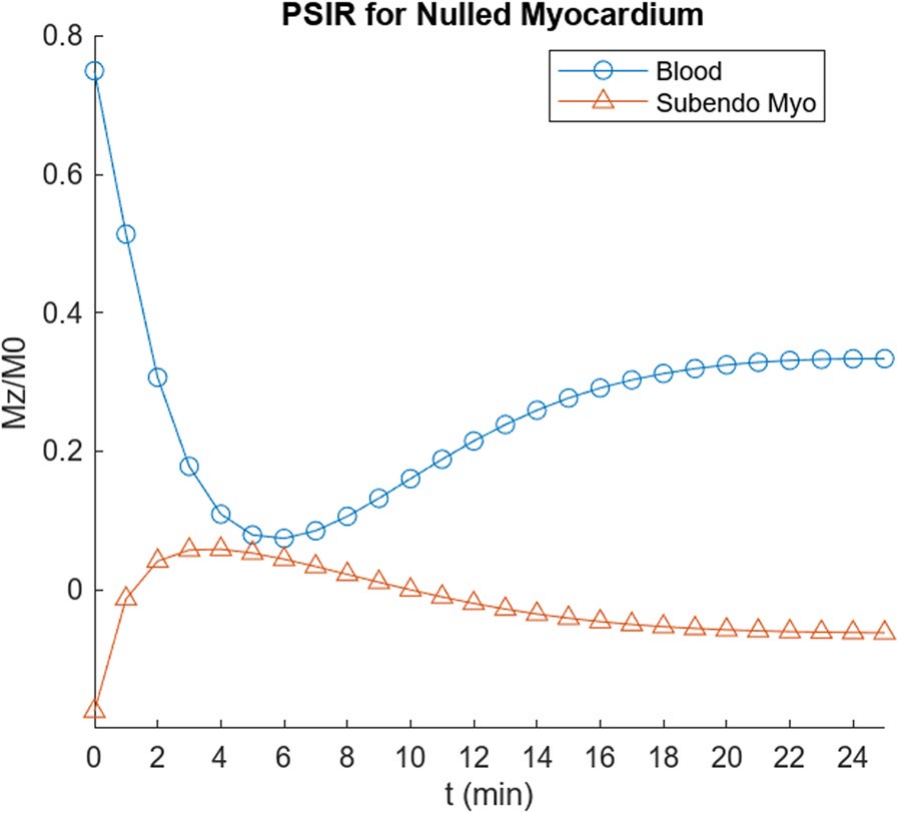
Calculated signal for PSIR imaging with nulling of reference myocardium, for simulated subendocardium in cardiac amyloid (parameter values as per Table 4), showing: (1) reduced signal from the blood and (2) reduced relative enhancement of the myocardium, compared with simulated amyloid scar in Fig. 8, consistent with clinical observations

For simulation of the LGE dynamics of cardiac amyloid, we first adjusted the value of V_b_ to better fit the initial values of the blood T1 data for the amyloid patients in [1], assuming an unchanged value of K, yielding an estimate of V_b_ = 0.115 (an increase of ~ 15% from the normal value). Keeping these values fixed, we then then further adjusted the value of V_e_ to better fit the full set of values of the amyloid blood T1, yielding an estimate of V_e_ = 0.13 (an increase of ~ 40% from the normal value), which reflects the expected generalized amyloid tissue deposition. To illustrate the sensitivity of the fitting process to the parameter values, blood T1 simulations for three values of V_e_ are shown in Fig. 9, together with the amyloid blood data extracted from Fig. 2 of [1]. To then simulate the enhancement of the myocardium in the presence of diffuse myocardial infiltration with amyloid (although with subendocardial predominance), we increased the reference (e.g., mid wall) myocardial EES, v_e_ (to ~ 0.75); and decreased the myocardial permeability-surface area product, PS (to ~ 0.32), as summarized in Table 4, with representative results as also shown in Figs. 10 and 11, together with cardiac amyloid blood and myocardial enhancement data extracted from Fig. 2 of [1].

**Table 4:** Modified parameter values used to simulate diffuse mid wall amyloid-infiltrated myocardial contrast kinetics

PS_m_	0.32 mL/min
v_em_	0.75
v_bm_	0.11
PS_s_	0.44 mL/min
v_es_	0.96
v_bs_	0.04
T1_m0_	1100 ms
V_b_	0.115
V_e_	0.13

Note that the T1 value of the blood in the cardiac amyloid simulation is now very close to that from the reference (“subepicardial”) myocardium (Fig. 10), with nulling of the reference myocardium signal resulting in low apparent signal from the blood in PSIR imaging (Fig. 11), as is observed clinically, as seen in Fig. 1. The relative degree of enhancement of the simulated amyloid subendocardial myocardium is decreased relative to that seen in the simulated scar LGE imaging, again, as is commonly observed clinically, although it is still brighter than the reference myocardium. These findings persist into the “pseudo steady-state” regime. Both the expanded body EES and the expanded reference myocardium EES are thus found to contribute significantly to the typical CMR appearance of cardiac amyloid.

### Modeling of atypical LGE appearance of cardiac amyloid

While we have demonstrated how a combination of expansion of the myocardial EES and the general body EES can jointly contribute to the characteristic MRI LGE appearance of cardiac amyloid, we can further use the LGE simulation program to investigate the likely causes of patients who are found to have the “characteristic” appearance of cardiac amyloid on MRI LGE imaging, without positive myocardial biopsy. In particular, any other conditions that would lead to similar changes in the size of the body or myocardial EES could have similar changes in the simulated contrast agent kinetics. We can also use the LGE simulation program to investigate the likely causes of cases where we fail to see the characteristic MRI LGE appearance in patients with a positive myocardial biopsy. In this case, this is likely just a reflection of the relative amount of amyloid deposition being too low to produce the degree of expansion of the body and myocardial EES needed to produce the “characteristic” MRI LGE appearance of cardiac amyloid, as is seen if we readjust the corresponding modified parameter values for cardiac amyloid to be closer to the initial values used for fitting normal enhancement dynamics.

## Discussion

Although the basic factors contributing to the dynamics of LGE in CMR have long been well understood, they have not previously been incorporated into a specific computer model of the myocardial contrast agent kinetics. This interactive model of the dynamics of myocardial contrast enhancement provides insight into the relative roles of different factors contributing to the qualitatively different kinetics of contrast enhancement seen in normal myocardium, scar, and cardiac amyloid, as well as some estimates of the values of the associated model parameters in these different cases. Although the model is necessarily a simplified representation of the underlying physiology, it captures some of the major features seen in clinical practice, and is able to approximately reproduce their expected quantitative scales. Although here we have focused on the use of the model for studying the dynamics of myocardial late gadolinium enhancement in cardiac amyloid, it would also potentially be readily adapted for modeling the contrast enhancement dynamics of other organs and other disease states.

Attempts to calculate specific values of the relatively large number of model parameters from curve fitting of the observed contrast kinetics are likely to be somewhat unreliable, due to the generally ill-posed nature of such “curve stripping” and the under-constrained situation in this case, with only 4 key parameters being potentially available from the biexponential plasma concentration curves and two more parameters from the triexponential tissue concentration curves. However, the systematic approach that we have taken here to the parameter fitting allows us to focus on the fitting of only a few parameters at a time, which helps to constrain and stabilize the fitting process.

The model was able to qualitatively reproduce the observed data on kinetics of contrast enhancement from [1], with reasonable values of the associated model parameters. In particular, it was able to fit the contrast enhancement dynamics of the blood and myocardium for the control subjects very well.

While we did not have comparable data on dynamic enhancement of myocardial fibrotic scar to work with, the model was able to capture the overall behavior of the endocardial enhancement in the cardiac amyloid patients, which would be expected to be qualitatively similar to other kinds of LGE. Interestingly, although the overall scale of the enhancement could be modeled well, the detailed shapes of the curves of the early and late phases of the subendocardial enhancement over time were not able to be fit as well, suggesting that the assumption of a “well-stirred” tissue interstitial space may not be fully adequate for modeling LGE dynamics in abnormal myocardium, as is discussed further below.

The principal aim of this work was to better understand the origins of the distinctive features of the LGE dynamics observed in many cases of cardiac amyloid: (1) diffuse (and somewhat weak) subendocardial LGE, and (2) a relatively dark appearance of the blood in PSIR images, through using the model for fitting of the contrast enhancement data in [1]. The modelling suggests that the known diffuse involvement of the mid wall of the myocardium with amyloid infiltration, although to a lesser extent than the infiltration of the subendocardium, can partially explain both of these features. However, the modeling suggests that the expanded body EES in amyloid also plays a role; the fitting of the altered blood enhancement dynamics suggests an expansion of this space on the order of 40%. Although there is no reliable independent way to check this value, this suggests that such a “tracer dilution” approach could provide a useful estimate of the total body amyloid burden, which would be an added benefit of the analysis of dynamic contrast enhancement in CMR of cardiac amyloid patients. The modeling suggests that the blood space is also increased in cardiac amyloid patients, although to a lesser degree (~ 15%) than the body EES.

Although the scale of the myocardial enhancement in cardiac amyloid could be modeled fairly well, the detailed shapes of the early and late phases of the myocardial enhancement were not able to be fit as well, suggesting that the assumption of a “well-stirred” tissue interstitial space may not be fully adequate for modeling LGE dynamics of abnormal myocardium with an expanded tissue EES. The role of diffusion of contrast agent within an expanded tissue interstitial space may not be fully captured by simply decreasing the effective value of the PS of the capillary-EES interface in the model. In particular, if the time scale for diffusion of contrast agent within the interstitium is on the order of or longer than the time scale for changes in the plasma concentration, rather than the EES effectively being a simple “well-stirred” compartment, spatial gradients of contrast agent concentration may develop within the interstitium, around the capillaries [23]. In the initial phases of contrast enhancement, when the interstitium is effectively acting as an integrator of the contrast agent entering the EES, this will not much affect the qualitative aspects of the contrast enhancement dynamics; the entry of the contrast agent into the interstitium from the plasma will still be primarily limited by the capillary permeability, and the effective size of the interstitial EES will be determined by the length scale of the diffusion process during this phase. However, in the later stages of contrast enhancement, with falling plasma concentrations, clearance of the contrast agent from the tissue will be slowed by the additional delay imposed by the need for contrast agent to diffuse back through the interstitium, before it can exchange across the capillary into the plasma. Thus, diffusion of contrast agent within an expanded tissue interstitium can potentially prolong the clearance of the contrast agent from the tissue, beyond what would be expected with a simple homogeneous EES. Contrast agent will diffuse radially away from the capillary within the interstitial space, at a rate determined by the diffusion coefficient, *D*, and the local concentration gradient, as per the Fick diffusion law [24]. If the size of the interstitial space is small enough and the diffusion of the contrast agent within it is fast enough, the interstitial space will be effectively “well-stirred” over the time scale of changes in the plasma concentration, with negligible concentration gradients. However, if the size of the interstitial space is too large for the contrast agent to diffuse well within it on this time scale, there may be significant concentration gradients within the interstitial space, which could effectively slow the clearance of the contrast agent from the tissue in the washout phase. The classical diffusion length over a time *t* is on the order of $2\sqrt{Dt};$ for a given diffusion distance, d, the corresponding diffusion time is on the order of d^2^/4*D*. Although we do not have a specific value for *D* of contrast agents like Gd-DTPA in myocardial interstitium, we can use an estimated value of *D* on the order of 2 × 10^–4^ mm^2^s^−1^, [23, 25, 26]; for an intercapillary distance of 20 microns, the diffusion time would then be on the order of a fraction of a second. However, for a diffusion distance of 200 microns, the diffusion time would be 100 times as long, or on the order of a minute. Steric hindrance of the contrast agent diffusion within the interstitial space by increased interstitial macromolecules (e.g., collagen or amyloid) would be expected to decrease the value of *D*, and thus to correspondingly increase the associated diffusion time and further prolong the contrast agent clearance phase. Replacing the simple “well-stirred” myocardial EES model with a catenary two-component model (Appendix 4) improves the agreement of the predicted and observed contrast enhancement dynamics.

Although the model was able to reproduce the distinctive features of LGE in cardiac amyloid well, not all patients with cardiac amyloid have these features in their CMR images. As suggested in [1], this is likely just the result of those patients having a relatively lower degree of expansion of the EES by amyloid, in both the myocardium and the body. On the other hand, some patients with CMR appearances suggesting the presence of cardiac amyloid turn out to have negative subsequent myocardial biopsies for amyloid. Aside from possible sampling error, where the distribution of amyloid deposition was heterogeneous and biopsies missed regions with significant amyloid, the factors that lead to the characteristic appearance may potentially also be found in some other conditions: (1) Although the control hypertensive patients in Maceira’s report [1] did not show any changes in their CMR appearance, there is a wide spectrum of the degree of fibrosis (and associated expected LGE) in pressure overload-associated left ventricular hypertrophy [27]. Thus, in the setting of diffuse fibrosis of the myocardium, nulling it (rather than a “true” reference myocardium) would tend to decrease the relative PSIR signal from the blood, leading to a relatively darker appearance of the blood. (2) Conditions other than amyloid, such as generalized edema (as might be associated with congestive heart failure), could potentially lead to an expanded body EES, which would contribute to decreased relative enhancement of the blood. (3) Other conditions than cardiac amyloid, such as generalized ischemia (such as might be associated with prolonged cardiac arrest), could lead to a relatively diffuse pattern of subendocardial fibrosis (and associated LGE), which could contribute to simulating the “characteristic” diffuse subendocardial LGE appearance of cardiac amyloid.

### Limitations

This study was performed retrospectively, using previously published data from patients who had had both conventional CMR and T1 mapping; thus, there may be associated selection biases in the results.

While we used a well-established three-compartment pharmacokinetic model of MRI contrast enhancement (originally applied to the brain) to calculate the myocardial contrast enhancement dynamics, there are simplifications associated with the model that could limit the reliability of the results, such as neglecting the possibility of diffusion-related concentration gradients in the tissue interstitial space, which is here assumed to be “well-stirred”. Extending the model to a catenary model of the myocardial EES can improve the results.

The presence of multiple potentially competing effects of the different underlying physiology changes, and the limited data available to characterize them, makes it difficult to assign levels of importance to the relative contribution of these different factors in the observed dynamics. However, we have used a systematic approach to the fitting of the model parameter values, to try to reduce the confounding effects of having multiple parameters to be fit.

The fitted parameter values were adjusted to match the observed enhancement dynamics of control (hypertensive) subjects rather than truly normal subjects. Thus, although they “had no other cardiovascular abnormality from the clinical history and examination or by CMR” [1], they may have had some associated fibrosis and myocyte hypertrophy relative to normal subjects, with potential associated effects on the values of v_e_ and v_mb_.

## Conclusion

A three-compartment model of the dynamics of myocardial contrast enhancement in magnetic resonance imaging is able to capture the qualitative features of late gadolinium enhancement (LGE), in both control subjects and patients with cardiac amyloid. In particular, the characteristic “dark blood” appearance of PSIR images of LGE in cardiac amyloid is seen to likely primarily reflect expansion of the extravascular extracellular space (EES) by amyloid in the “reference” myocardium. The altered cardiac amyloid contrast kinetics also reflect expansion of the body EES.

### Supplementary Information

**Additional file 1.** LGE_sim_catenary.mlapp. MATLAB program for interactive simulation of contrast enhancement dynamics.

## Figures and Tables

**Fig. 12:**
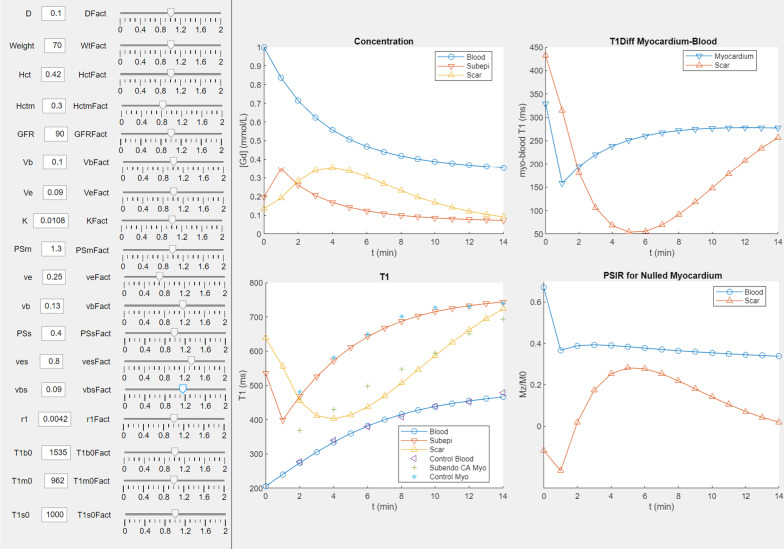
Representative display of user interface of interactive program used for simulation of contrast enhancement kinetics (Appendix 2), incorporating 3-compartment model in Fig. 2. Default values for different model parameters can be entered in windows at left and scaled with adjacent interactive sliders. Resulting calculated variables are displayed in the graphs at the right, including: serial concentrations of contrast agent in blood, reference myocardium and “scar” or amyloid deposits (top left); corresponding serial T1 values (bottom left); serial T1 differences of myocardium with blood (top right); and serial longitudinal magnetization values (proportional to signal) for PSIR imaging set to null reference myocardium (bottom right). Some T1 data extracted from Fig. 2 of [1] are also displayed, for comparison with model results

**Fig. 13:**
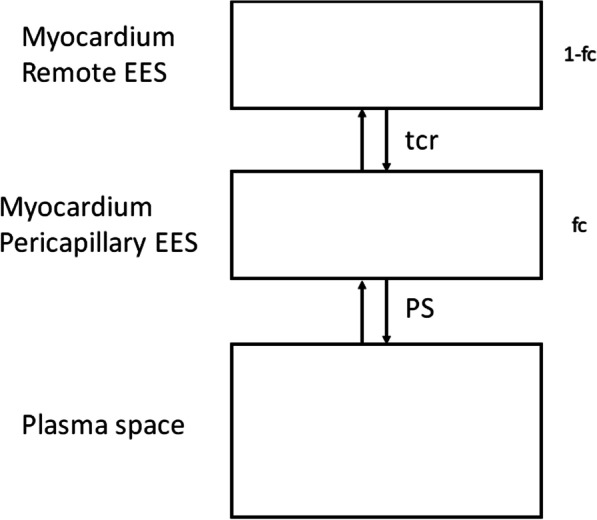
Catenary compartment model of contrast agent exchange in expanded myocardium EES, with pericapillary and remote components. The pericapillary component has fractional volume f_c_; the remote component has fractional volume (1-f_c_). The pericapillary compartment exchanges with both the plasma space and the remote compartment; the remote compartment only exchanges with the pericapillary compartment, at a rate dependent on the effective exchange time constant, t_cr_

**Fig. 14:**
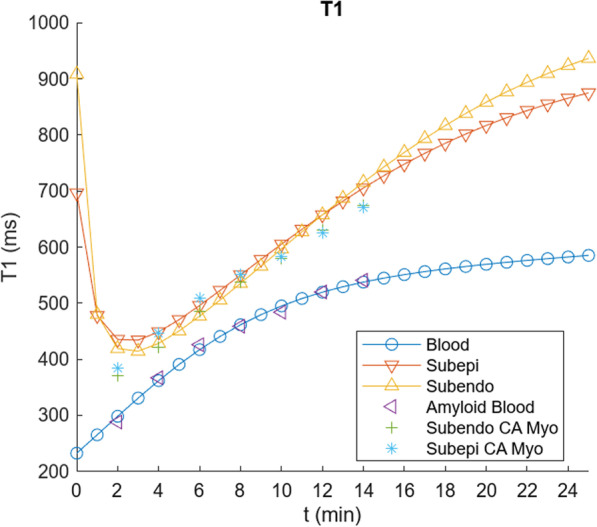
Repeat calculations of T1 as in Fig. 10, with catenary model for myocardial EES, showing better agreement with observational data

## Data Availability

Not applicable (using previously published data). A link is provided to download the software used.

## Appendix 1

### Mathematical model of LGE kinetics

The contrast agent kinetics can be modeled with the following equations, based on the analysis of Tofts and Kermode [9]; we will primarily follow the standard recommendations for denoting quantities and symbols related to contrast-enhanced dynamic MRI [28].

The initial plasma concentration, C_p_, at time t = 0 after a bolus dose per unit body weight of contrast agent, D, will be given (after initial mixing into the plasma space) by

1 $${\text{C}}_{{\text{p}}} \left( 0 \right) \, = {\text{ D}}/{\text{V}}_{{\text{p}}} ,$$

where V_p_ is the volume of the plasma per unit body weight; for a given hematocrit, Hct, V_p_ is related to the total blood volume per unit body weight, V_b_, by

2 $${\text{V}}_{{\text{p}}} = \, \left( {{1} - {\text{Hct}}} \right){\text{ V}}_{{\text{b}}}$$

The rate of clearance of contrast agent from the plasma by the kidneys will be determined by the product of C_p_ and the GFR per unit body weight. The rate of exchange of contrast agent between the plasma and the total body EES will be determined by the product of the difference in their contrast agent concentrations (C_p_ and C_e_, respectively) and the total permeability coefficient of the capillaries between them per unit body weight, K. The net rates of change in the concentrations in the plasma and the total body EES will thus depend on K and the volumes of distribution per unit body weight of the contrast agent in the plasma and the total body EES (V_p_ and V_e_, respectively), as well as the GFR:

3 $${\text{dC}}_{{\text{p}}} /{\text{dt}} = \, \left( { - {\text{ C}}_{{\text{p}}} {\text{GFR }} + {\text{ K}} \left( {{\text{C}}_{{\text{e}}} {-}{\text{ C}}_{{\text{p}}} } \right)} \right)/{\text{ V}}_{{\text{p}}} ,$$

4 $${\text{dC}}_{{\text{e}}} /{\text{dt}} = \, \left( {{\text{K}} \left( {{\text{C}}_{{\text{p}}} {-}{\text{ C}}_{{\text{e}}} } \right)} \right)/{\text{ V}}_{{\text{e}}} .$$

The corresponding net blood concentration, C_b_, can be calculated from C_p_ and the hematocrit, Hct:

5 $${\text{C}}_{{\text{b}}} = \, \left( {{1} - {\text{Hct}}} \right){\text{ C}}_{{\text{p}}}$$

Similarly, the exchange of contrast agent between the plasma and the myocardial EES will be determined by the product of the difference in their contrast agent concentrations (C_p_ and C_me_, respectively) and the permeability-surface area product per unit tissue volume of the capillaries between them, PS, with an associated rate of change in the concentration of the myocardial EES that depends on PS and the fractional volume of the myocardial EES, v_e_,

6 $${\text{dC}}_{{{\text{me}}}} /{\text{dt}} = \, \left( {{\text{PS}} \left( {{\text{C}}_{{\text{p}}} {-}{\text{ C}}_{{{\text{me}}}} } \right)} \right)/{\text{ v}}_{{\text{e}}}$$

Thus, for a given set of values for these various parameters, we can calculate the corresponding time-dependent changes of the concentrations.

With a few reasonable assumptions, including that the plasma space is more strongly coupled to the interstitium than to the kidneys, the time course of the plasma concentration with this model is found to be a biexponential curve [6]:

7 $${\text{C}}_{{\text{p}}} \left( {\text{t}} \right) \, = {\text{ D }}\left\{ {{\text{a}}_{{1}} {\text{exp}}\left( { - {\text{m}}_{{1}} {\text{t}}} \right) \, + {\text{ a}}_{{2}} {\text{exp}}\left( { - {\text{m}}_{{2}} {\text{t}}} \right)} \right\},$$

with the fast component (a_1_, m_1_) reflecting equilibration of the contrast agent between the plasma and the body interstitial space, and the slow component (a_2_, m_2_) reflecting renal clearance of the contrast agent from the plasma and the interstitial space. The variables of the biexponential curve are defined in terms of the other model parameters as follows:

8 $${\text{a}}_{{1}} = {\text{ V}}_{{\text{e}}} /\left( {{\text{V}}_{{\text{p}}} \left( {{\text{V}}_{{\text{p}}} + {\text{ V}}_{{\text{e}}} } \right)} \right) \, ;{\text{ a}}_{{2}} = { 1}/\left( {{\text{V}}_{{\text{p}}} + {\text{ V}}_{{\text{e}}} } \right)$$

9 $${\text{m}}_{{1}} = {\text{ K}}\left( {{\text{V}}_{{\text{p}}} + {\text{ V}}_{{\text{e}}} } \right)/{\text{V}}_{{\text{p}}} {\text{V}}_{{\text{e}}} ;{\text{ m}}_{{2}} = {\text{ GFR}}/\left( {{\text{V}}_{{\text{P}}} + {\text{ V}}_{{\text{e}}} } \right)$$

The corresponding myocardial EES contrast agent concentration over time is then a triexponential curve:

10 $${{\text{C}}_{{\text{me}}}} = {\text{D}}\left\{ {\frac{{{m_3}{a_1}}}{{{m_3} - {m_1}}}{\text{exp}}\left( { - {m_1}{\text{t}}} \right){\mkern 1mu} + \frac{{{m_3}{a_2}}}{{{m_3} - {m_2}}}{\text{exp}}\left( { - {m_2}{\text{t}}} \right){\mkern 1mu} + {\text{ c exp}}\left( { - {m_3}{\text{t}}} \right)} \right\}$$

where m_3_ = PS/v_e_, and c is a constant that can be determined from the initial condition, C_me_ (0) = 0.

The predicted biexponential behavior of the plasma contrast agent concentration has been confirmed experimentally. For labeled DTPA studies by Weinmann [29], Tofts and Kermode [9] found typical normal values for the plasma contrast-time curve parameters of: a_1_ = 3.99 kg/L, a_2_ = 4.78 kg/L, m_1_ = 0.144 min^−1^, and m_2_ = 0.0111 min^−1^.

The corresponding net myocardial concentration, C_m_, (reflecting contributions from both interstitial space and plasma) can be calculated from C_me_ and the fractional plasma volume of the myocardium, v_p_, which can, in turn, be calculated from the fractional blood volume of the myocardium, v_b_, along with the tissue hematocrit, Hct_m_:

11 $${\text{C}}_{{\text{m}}} = {\text{ v}}_{{\text{e}}} {\text{C}}_{{{\text{me}}}} + {\text{ v}}_{{\text{b}}} \left( {{1} - {\text{Hct}}_{{\text{m}}} } \right){\text{ C}}_{{\text{p}}}$$

The tissue microcirculation hematocrit may be smaller than the large vessel hematocrit, due to the Fåhræus effect [30, 31]; for example, Kunze et al. found a myocardial tissue hematocrit of 0.26 at rest and 0.37 at adenosine stress, when comparing PET- and MRI-derived myocardial perfusion studies [32].

Assuming that the relaxivity, r1, of the contrast agent is the same in blood and myocardium [21], and that the intracellular and extracellular water are in rapid exchange with the contrast agent, we can then calculate the corresponding relaxation rates of the blood and the myocardium, R1_b_ and R1_m_, respectively:

12 $${\text{R1}}_{{\text{b}}} =_{ } {\text{R1}}_{{{\text{b}}0}} +_{ } {\text{r1 C}}_{{\text{b}}} ,$$

and

13 $${\text{R1}}_{{\text{m}}} =_{{}} {\text{R1}}_{{{\text{m}}0}} +_{{}} {\text{r1 C}}_{{\text{m}}} ,$$

where R1_b0_ and R1_m0_are the corresponding relaxation rates in the absence of contrast agent. Thus, we can finally calculate the T1 relaxation times for blood and myocardium, T1_b_ and T1_m_, respectively:

14 $${\text{T1}}_{{\text{b}}} = {1}/{\text{ R1}}_{{\text{b}}} ,$$

and

15 $${\text{T1}}_{{\text{m}}} = {1}/{\text{ R1}}_{{\text{m}}} .$$

## Appendix 2

### Interactive computer model of LGE dynamics in CMR

The equations for contrast agent concentrations over time after a bolus injection of a dose of contrast agent into the plasma space of a 3-compartment model, given in Appendix 1, were used to create an interactive program with MATLAB (Mathworks, Natick, MA). For a given set of model parameter values, the program displays: (1) the resulting serial concentrations of contrast agent in the blood and the myocardium, along with (2) the corresponding T1 values, (3) the difference in T1 between the myocardium and the blood, and (4) the signal with PSIR imaging when the inversion time has been adjusted to null the reference myocardium signal (as described in Appendix 3). A representative display from the program is shown in Fig. 12.

## Appendix 3

### Mathematical model of PSIR imaging of LGE kinetics

The image intensity at a time, TI, after an inversion pulse will be proportional to the longitudinal magnetization Mz; for starting at an equilibrium magnetization, M0, we have

16 $${\text{Mz}}/{\text{M}}0 \, \left( {{\text{TI}}} \right) \, = { 1} - {\text{2 exp }}\left( { - {\text{TI}}/{\text{T1}}} \right).$$

In order to bring out areas in the image of T1 shortening due to relatively increased contrast agent concentration, TI is usually empirically chosen such that the magnetization of the reference normal myocardium (or the myocardial regions visually least affected by abnormal contrast enhancement) will be near zero:

17 $${\text{TI}} = {\text{ T1}}_{{\text{m}}} {\text{ln2}}.$$

Using this value for the TI, together with Eq. 16, will let us calculate the corresponding PSIR signal for the blood (or for more greatly contrast-enhanced portions of the heart wall) when the inversion pulse timing has been adjusted so as to null the reference myocardium signal.

## Appendix 4

### Catenary model of myocardial EES compartment

As an approximate model of the inhomogeneous concentrations resulting from increased diffusion times within an expanded myocardial EES, we can represent the myocardial EES as a catenary model composed of two components: (1) a pericapillary EES component, with fractional volume f_c_, exchanging with both the plasma space and a more remote component, and (2) a remote EES component, with fractional volume (1-f_c_), exchanging only with the pericapillary EES component (Fig. 13). The contrast concentration in the pericapillary component will be determined by exchange with the plasma space concentration, which can be modelled as above; if the exchange with the remote component is relatively slow, its effect on the pericapillary component concentration can be approximately neglected. The rate of change of the concentration of the remote component will depend on the concentration difference between the components; it will also depend on the ratio of the effective exchange coefficient between the components and the volume of the remote component. This will determine an equivalent time constant, t_cr_, for the change in the remote component concentration. Thus, given values for the fractional volume of the pericapillary component and the time constant for exchange between the pericapillary and remote components, we can calculate the remote component concentration over time by integrating its rate of change, e.g., with a simple Euler forward difference approach. For f_c_ = 1, this model reduces to the simpler three-compartment model above. The MATLAB program for an interactive app implementation of the catenary model is included as Additional file 1. Use of the catenary model with the same other parameter values as used in Fig. 10 can produce a better fit to the observed data points from (1), as shown in Fig. 14 with f_c_ = 0.5 and t_cr_ = 8 s.

## Abbreviations

C_b_: Blood concentration
C_e_: Body EES concentration
C_m_: Net myocardial concentration
C_me_: Myocardial EES concentration
C_p_: Plasma concentration
CMR: Cardiac magnetic resonance imaging
d: Diffusion distance
D: Dose
*D*: Diffusion coefficient
EES: Extravascular extracellular space
f_c_: Fractional volume of myocardium EES pericapillary component
GFR: Glomerular filtration rate
Hct: Hematocrit
Hct_m_: Tissue hematocrit
K: Permeability coefficient of the body capillaries per unit body weight
LGE: Late gadolinium enhancement
LA: Left atrium
LV: Left ventricle
M0: Equilibrium magnetization
Mz: Longitudinal magnetization
MRI: Magnetic resonance imaging
PET: Positron emission tomography
PS: Permeability-surface area product of the capillaries per unit tissue volume
PSIR: Phase-sensitive inversion-recovery
PS_m_: PS of the myocardium
r1: Relaxivity
R1_b_: Relaxation rate of the blood
R1_b0_: Relaxation rate of the blood without contrast agent
R1_m_: Relaxation rate of the myocardium
R1_bm0_: Relaxation rate of the myocardium without contrast agent
RA: Right atrium
RV: Right ventricle
SAP: Serum amyloid P component
T: Diffusion time
t_cr_: Time constant for change of concentration in myocardium EES remote component
TI: Time after an inversion pulse
T1_b_: T1 relaxation time for blood
T1_m_: T1 relaxation time for myocardium
v_b_: Fractional blood volume of the myocardium
v_bs_: Fractional blood volume of the scar myocardium
v_e_: Fractional volume of the myocardial EES
v_es_: Fractional volume of myocardial scar EES
v_p_: Fractional plasma volume of the myocardium
V_b_: Blood volume per unit body weight
Ve: EES volume per unit body weight
V_p_: Plasma volume per unit body weight
