# Supplementary figures and images for: Modeling of factors affecting late gadolinium enhancement kinetics in MRI of cardiac amyloid
Source: J Cardiovasc Magn Reson. 2023 Aug 10;25:46. doi: 10.1186/s12968-023-00952-x (PMC10413700; doi:10.1186/s12968-023-00952-x)

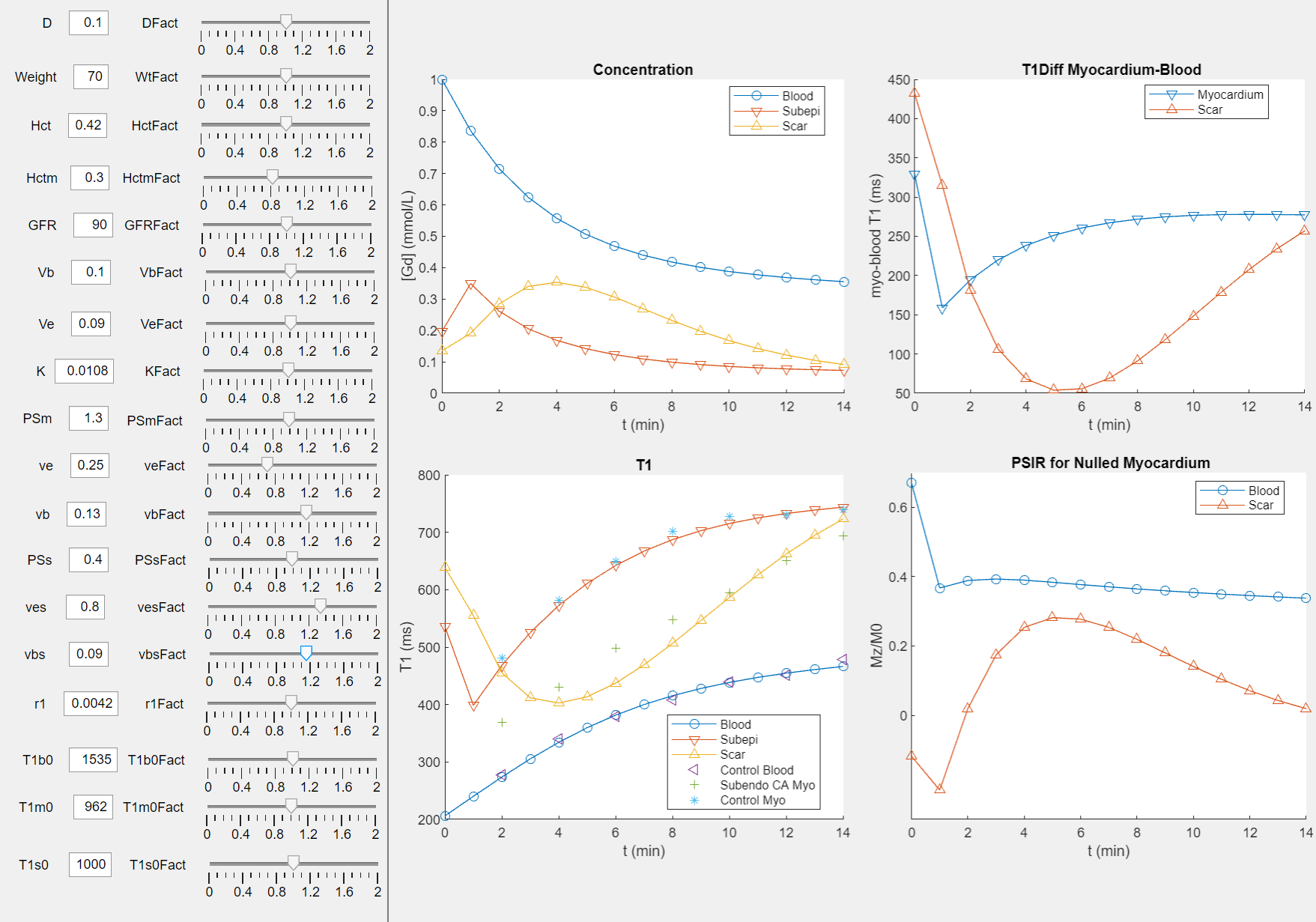

Supplement: Supplementary file 1 — Additional file 1. LGE_sim_catenary.mlapp. MATLAB program for interactive simulation of contrast enhancement dynamics. [file 12968_2023_952_MOESM1_ESM.mlapp › metadata/appScreenshot.png]
